# Impact of extended-spectrum chromosomal AmpC (ESAC) of *Escherichia coli* on susceptibility to cefiderocol

**DOI:** 10.1128/spectrum.00704-24

**Published:** 2024-06-11

**Authors:** Otávio Hallal Ferreira Raro, Christophe Le Terrier, Patrice Nordmann, Laurent Poirel

**Affiliations:** 1 Medical and Molecular Microbiology, Faculty of Science and Medicine, University of Fribourg, Fribourg, Switzerland; 2 Division of Intensive Care Unit, University hospitals of Geneva, Geneva, Switzerland; 3 Swiss National Reference Center for Emerging Antibiotic Resistance (NARA), University of Fribourg, Fribourg, Switzerland; Instituto de Higiene, Montevideo, Canelones, Uruguay

**Keywords:** AmpC, *Escherichia coli*, ESAC, cefiderocol

## Abstract

The impact of chromosomally encoded wild-type or extended-spectrum (ESAC) AmpC β-lactamases of *Escherichia coli* on susceptibility to ceftazidime, cefepime, and cefiderocol was evaluated in different genetic backgrounds, including wild-type, PBP3-modified, and porin-deficient *E. coli* strains. Recombinant *E. coli* strains possessing the different backgrounds and producing variable ESACs were evaluated. Although ESAC enzymes conferred resistance to ceftazidime and decreased susceptibility to cefepime as expected, we showed here that cefiderocol was also a substrate of ESAC enzymes.

**IMPORTANCE:**

We showed here that chromosomally encoded intrinsic extended-spectrum cephalosporinases of *Escherichia coli* may impact susceptibility not only to ceftazidime and cefepime but also to cefiderocol.

## OBSERVATION

*Escherichia coli* is the most common pathogen intrinsically susceptible to most antibiotics and particularly to β-lactams (1). It may cause either community or nosocomial infections (1, 2). Although acquired resistance to broad-spectrum cephalosporins in that species may be related to the acquisition of expanded-spectrum β-lactamases, it may also be related to the production of the intrinsic chromosomally encoded AmpC (named cAmpC) of *E. coli* where the gene is usually not expressed or at very low level (3).

In *E. coli*, the expression of the cAmpC gene is constitutive, and differently from the other Enterobacterales species possessing an intrinsic AmpC-encoding gene, it is not associated to any *ampR-*type regulatory gene, encoding a transcriptional regulator repressing the expression of the *ampC* gene at basal levels (3). The cAmpC production is regulated by promoter/attenuator mechanisms that commonly end up in low levels of expression (4). However, it has been shown that mutations in the promoter region of the intrinsic *ampC* gene of *E. coli* might enhance the expression of that gene, therefore leading to acquired resistance to broad-spectrum cephalosporins (5, 6). High-level production of cAmpCs has therefore been shown to be a potential source of such acquired resistance (3).

AmpCs are usually active against cephalosporins (i.e., cephamycins) and aztreonam at variable levels. However, they do not possess significant hydrolytic activities against fourth-generation cephalosporins such as cefepime or cefpirome, as well as against carbapenems. AmpC enzymes are resistant to the β-lactamase inhibitors sulbactam, clavulanate, and tazobactam but are susceptible to avibactam (3, 7, 8).

Nevertheless, some variants of those cAmpCs possess the capacity to confer reduced susceptibility to third- but also fourth-generation cephalosporins once produced at a high level (3, 5). These specific variants have been defined as extended-spectrum AmpC β-lactamases (ESAC) (9, 10), and they do possess increased catalytic activity against oxyimino-cephalosporins, including cefepime and cefpirome. Noteworthy, ESAC enzymes have been identified in other Gram-negative species, including *Enterobacter cloacae*, *Serratia marcescens*, *Pseudomonas aeruginosa*, and *Acinetobacter baumannii* (11–14). ESAC enzymes, compared with classical AmpC enzymes, possess structural modifications that may occur in six different regions of the protein, being all in the vicinity of the active site, and corresponding either to the omega loop, the H-2 helix, the H-9 helix, the H-10 helix, the R2 helix, or the H-11 helix (15, 16).

The objective of the present study was to evaluate whether ESAC produced by some *E. coli* isolates might be interfering with the activity of the newly developed siderophore cephalosporin cefiderocol, considering the structural similarities between cefiderocol and ceftazidime, but also to some extent cefepime and cefpirome.

The isolates were available in the collection of the Laboratory of Medical and Molecular Microbiology (Fribourg, Switzerland) and were formerly obtained for a previous study published by our group (10). The genes encoding the different cAmpC were from different *E. coli* clinical isolates, with six isolates producing ESACs as defined previously (10), namely, AmpC-EC13, -EC14, -EC15, -EC16, -EC17, and -EC18, and a single isolate producing a narrow-spectrum AmpC, namely, AmpC-EC5.

PCR amplification of the cAmpC genes was performed using primers described by Mammeri et al. (10). A Sanger sequencing (Microsynth, Balgach, Switzerland) followed by sequence analyses using MultAlin tool (17) was performed to confirm the presence and the variant of the *ampC* genes. After that, the cAmpCs were transformed into *E. coli* TOP10 using the pCR-Blunt II-TOPO vector (Invitrogen, Waltham, MA, USA) and selection was performed in Luria-Bertani (LB, Carl-Roth, Karlsruhe, Germany) agar plates either with kanamycin (Sigma-Aldrich, St. Louis, MO, USA) 50 mg/L or kanamycin 50 mg/L plus amoxicillin (Sigma-Aldrich) 30 mg/L. Plasmids were extracted with the Zyppy Plasmid Miniprep Kit (Zymo Research, Irvine, CA, USA) and subsequently transformed in *E. coli* MG1655 and its counterpart *E. coli* MG1655 exhibiting modifications in its PBP3 penicillin-binding protein, corresponding to amino acid insertion YRIN (Tyr-Arg-Ile-Asn) or YRIK (Tyr-Arg-Ile-Lys) (18). Those recombinant strains were elaborated with the objective of evaluating the impact of ESACs in PBP3-modified *E. coli* backgrounds, since recent works highlighted their current circulation enhancing the β-lactam resistance profiles, especially to aztreonam and aztreonam-avibactam (18, 19). Additionally, recombinant plasmids were also electro-transformed into the porin-deficient *E. coli* HB4 (lacking both OmpC and OmpF porins) to evaluate the interplay between ESAC production and permeability defects. All *E. coli* recipients strains used in this study did not express their intrinsic cAmpC.

Broth microdilution was performed to obtain the minimum inhibitory concentrations (MICs) of ceftazidime (Thermo Fisher Scientific, Waltham, MA, USA), cefepime (Merck, Darmstadt, Germany), and cefiderocol (Shionogi, Osaka, Japan), following the EUCAST guidelines (20, 21).

MIC results obtained for the *E. coli* parental and recombinant strains are shown in Table 1. As expected, all parental strains (TOP10, MG1655, MG1655 + PBP3insYRIN, MG1655 + PBP3insYRIK, and HB4) exhibited low MIC values to ceftazidime, cefepime, and cefiderocol. As expected, modifications of the PBP3 sequence of strain MG1655 had a significant effect on the MICs of ceftazidime and cefepime, with MICs increasing from 0.25 to 1 mg/L (4-fold) and ≤0.125 to 1 mg/L (eightfold), respectively, for both MG1655 + PBP3insYRIN and MG1655 + PBP3insYRIK when compared with the wild-type MG1655. By contrast, no impact on the MICs of cefiderocol w observed upon modifications of the PBP3 sequence.

**TABLE 1:** MICs (µg/mL) of cephalosporins for *E. coli* strains and recombinant *E. coli* strains producing narrow- or extended-spectrum chromosomal AmpC β-lactamases (ESAC)^*b*^

*E. coli* strain	AmpC^*c*^	Amino acid substitutions^*a*^	Ceftazidime	Cefepime	Cefiderocol
ATCC 25922	–	NA	≤0.125	≤0.125	0.5
TOP10	–	NA	≤0.125	≤0.125	≤0.06
TOP10 + pTOPO + EC5	Narrow	NA	4	≤0.125	≤0.06
TOP10 + pTOPO + EC13	ESAC	S287N	**128**	4	**4**
TOP10 + pTOPO + EC14	ESAC	V298L	**128**	4	**4**
TOP10 + pTOPO + EC15	ESAC	R296P	**>128**	**8**	**4**
TOP10 + pTOPO + EC16	ESAC	S287C	**64**	1	1
TOP10 + pTOPO + EC17	ESAC	R296P	**>128**	4	**4**
TOP10 + pTOPO + EC18	ESAC	S287N	**>128**	**8**	**4**
MG1655	–	NA	0.25	≤0.125	0.5
MG1655 + pTOPO + EC5	Narrow	NA	**8**	≤0.125	0.5
MG1655 + pTOPO + EC13	ESAC	S287N	**64**	1	**4**
MG1655 + pTOPO + EC14	ESAC	V298L	**64**	4	**4**
MG1655 + pTOPO + EC15	ESAC	R296P	**64**	2	**4**
MG1655 + pTOPO + EC16	ESAC	S287C	**64**	1	1
MG1655 + pTOPO + EC17	ESAC	R296P	**64**	2	**4**
MG1655 + pTOPO + EC18	ESAC	S287N	**64**	1	**4**
MG1655-YRIK	–	NA	1	1	0.5
MG1655-YRIK + pTOPO + EC5	Narrow	NA	**16**	1	0.5
MG1655-YRIK + pTOPO + EC13	ESAC	S287N	**>128**	**32**	**4**
MG1655-YRIK + pTOPO + EC14	ESAC	V298L	**64**	**32**	**4**
MG1655-YRIK + pTOPO + EC15	ESAC	R296P	**>128**	**64**	**8**
MG1655-YRIK + pTOPO + EC16	ESAC	S287C	**>128**	**8**	2
MG1655-YRIK + pTOPO + EC17	ESAC	R296P	**>128**	**64**	**4**
MG1655-YRIK + pTOPO + EC18	ESAC	S287N	**>128**	**8**	**4**
MG1655-YRIN	–	NA	1	1	0.5
MG1655-YRIN + pTOPO + EC5	Narrow	NA	**16**	1	0.5
MG1655-YRIN + pTOPO + EC13	ESAC	S287N	**>128**	**32**	**4**
MG1655-YRIN + pTOPO + EC14	ESAC	V298L	**>128**	**32**	**8**
MG1655-YRIN + pTOPO + EC15	ESAC	R296P	**>128**	**32**	**8**
MG1655-YRIN + pTOPO + EC16	ESAC	S287C	**>128**	**8**	2
MG1655-YRIN + pTOPO + EC17	ESAC	R296P	**128**	**16**	**4**
MG1655-YRIN + pTOPO + EC18	ESAC	S287N	**>128**	**8**	**4**
HB4	–	NA	0.5	0.5	0.25
HB4 +pTOPO + EC5	Narrow	NA	**16**	0.5	0.25
HB4 +pTOPO + EC13	ESAC	S287N	**>128**	**64**	**4**
HB4 +pTOPO + EC14	ESAC	V298L	**>128**	**16**	**4**
HB4 +pTOPO + EC15	ESAC	R296P	**>128**	**16**	2
HB4 +pTOPO + EC16	ESAC	S287C	**>128**	**16**	1
HB4 +pTOPO + EC17	ESAC	R296P	**>128**	**64**	**4**
HB4 +pTOPO + EC18	ESAC	S287N	**>128**	**16**	2

^*a*^ Amino acid substitutions corresponding to ESACs comparing with the narrow-spectrum cAmpC-EC5 and based on the structural alignment-based numbering of class C β-lactamase (SANC).

^*b*^ Bold, resistant; underlined, intermediate; normal script, susceptible; NA, not applicable.

^*c*^ Absence of AmpC enzyme is indicated with the – symbol.

Regardless of the genetic background, all recombinant strains producing the narrow-spectrum AmpC-EC5 showed an increase in their respective MICs of ceftazidime, ranging from 4 to 16 mg/L, corresponding to a 16- to 32-fold increase compared with their parental isogenic strains, but no increased effect was observed for either cefepime or cefiderocol.

Once producing the different ESAC enzymes, and as expected, the different *E. coli* TOP10 recombinant strains exhibited significantly increased MICs of ceftazidime, ranging from 64 to >128 mg/L compared with ≤0.125 MIC for the TOP10 parental strain, respectively. In addition, MICs of cefepime were also increased, ranging from 1 to 8 mg/L compared with ≤0.125 for the parental strain, respectively. This also concurs with previous studies showing that ESACs of *E. coli* exhibited hydrolytic activity toward cefepime (10). Interestingly, when evaluating susceptibility to cefiderocol, increased MIC values were observed for all the recombinant strains, being at 4 mg/L for all recombinant strains, except for the one producing AmpC-EC16 (MIC at 1 mg/L), while the MIC of the parental strain is ≤0.06 mg/L.

The same results were observed when using *E. coli* MG1655 as background, again showing that production of AmpC-EC16 had a less marked effect on susceptibility to cefepime and cefiderocol than the other variants. Overall, when comparing MG1655 alone from the modified MG1655 + PBP3insYRIK strain, significantly increased MIC values were observed upon production of ESAC enzymes. Almost identical values were obtained when comparing MG1655 + PBP3insYRIK to MG1655 + PBP3insYRIN, therefore suggesting that the type of insertion within the PBP3 sequence did not have impact on the MIC values, as previously suggested (8, 18, 22).

Finally, when using the HB4 porin-deficient strain as background, significant MIC increases were again observed when producing the different cAmpCs showing an increase in MIC values to cefiderocol and with AmpC-EC16 again showing the weaker impact (Table 1). Interestingly, in contrast with cefepime, the porin deficiency did not affect the MIC values for cefiderocol, suggesting that only the production of ESACs influenced the MIC results.

Table 1 shows all amino acid substitutions those cAmpCs have in comparison with the narrow-spectrum cAmpC-EC5 based on the SANC (23). All those substitutions (S287N, V298L, R296P, and S287C) confer ESAC proprieties to the cAmpC-ECs, according to our former study (10) and confirmed in the present study. We speculate that the amino acid substitution S287C present in AmpC-EC16 may provide a weaker response toward the cephalosporins hydrolysis when compared with the other amino acid substitutions (Table 1).

The recent development of cefiderocol offers a promising alternative for treating infections caused by multidrug-resistant Gram-negative infections. Despite sharing significant similarities in terms of structure with ceftazidime, cefiderocol possesses an additional C-7 side chain enhancing its stability against β-lactamases and an additional C-3 side chain preventing its recognition as substrate by several β-lactamases and in particular metallo-β-lactamases (24). Nevertheless, this similarity in terms of structure with ceftazidime explains why one might observe some collateral effect on cefiderocol once a given enzyme evolves toward increased activity against ceftazidime. This is, for example, the case with some mutants of the class A KPC-type β-lactamases conferring resistance to ceftazidime-avibactam (25, 26). Most of those variants do not exhibit a major reduction of their sensitivity toward avibactam, but rather a major increase in terms of binding to ceftazidime eventually leading to resistance to ceftazidime-avibactam. Hence, those KPC variants conferring resistance to ceftazidime-avibactam have been shown to confer increased MICs of cefiderocol (27).

Therefore, knowing that some AmpC-type β-lactamases may possess increased activity toward ceftazidime (defining the ESACs), our objective was to evaluate the ability of those ESAC to confer reduced susceptibility to cefiderocol. Our results showed that ESACs indeed exhibited increased activity toward cefiderocol and potentially constitute sources of reduced susceptibility to the novel cephalosporin in *E. coli*. The significant contribution of chromosomal ESACs in reduced susceptibility to cefiderocol in *E. coli* was systematically demonstrated, regardless of the background of the *E. coli*-recipient strains (wild type, altered in their PBP3, or altered in their permeability). Our data therefore identify an additional pathway potentially affecting susceptibility to cefiderocol in *E. coli*.

## Data Availability

All data from this study can be made available upon request, without limitation in time.
